# ZnO and MXenes as electrode materials for supercapacitor devices

**DOI:** 10.3762/bjnano.12.4

**Published:** 2021-01-13

**Authors:** Ameen Uddin Ammar, Ipek Deniz Yildirim, Feray Bakan, Emre Erdem

**Affiliations:** 1 Faculty of Engineering and Natural Sciences, Sabanci University, Tuzla 34956, Istanbul, Turkey; 2 Sabanci University Nanotechnology Research Centre (SUNUM), Sabanci University TR-34956 Istanbul, Turkey

**Keywords:** 2D materials, electrodes, MXenes, supercapacitors, zinc oxide (ZnO)

## Abstract

Supercapacitor devices are interesting owing to their broad range of applicability from wearable electronics to energy storage in electric vehicles. One of the key parameters that affect the efficiency of supercapacitor devices is selecting the ideal electrode material for a specific application. Regarding this, recently developed metal oxides, specifically nanostructured ZnO, and MXenes with their defect structures, size effects, as well as optical and electronic properties have been presented as electrode material in supercapacitor devices. The discussion of MXenes along with ZnO, although different in chemistry, also highlights the differences in dimensionality when it comes to defect-driven effects, especially in carrier transport. The volume under the influence of the defect centers is expected to be different in bulk and 2D structures, regardless of composition. Hence, analysis and discussion of both materials provide a fundamental understanding regarding the manner in which 2D structures are impacted by defects compared to bulk. Such an approach would therefore serve the scientific community with the material design tools needed to fabricate the next generation of supercapacitor devices.

## Introduction

In this article, the past, the present, and the prospects of ZnO and MXenes are discussed in terms of their usage as electrode materials in supercapacitor devices. Recently, supercapacitors gained a lot of attention due to their high power density as well as due to the potential to further increase the energy density. Supercapacitors may act as batteries in electrochemical performance tests. The choice of the materials, their morphology, dimension, and synthesis technique, as well the synergy with the other components of the supercapacitor device are important factors in this scenario. Nowadays, ZnO as metal oxide and MXene as 2D materials are the rising stars of electrode materials in supercapacitors due to their highly controllable properties. Therefore, we review the findings about ZnO and MXene in terms of defect structures and optical and electrical properties. This review will give a prospect to researchers working on the development of electrode materials for efficient supercapacitors.

The discussion of MXenes along with ZnO, although different in chemistry, also highlights the differences in dimensionality when it comes to defect-driven effects, especially in carrier transport. The volume under the influence of the defect centers is expected to be different in bulk and 2D structures, regardless of composition, Hence, analysis and discussion of both materials provide a fundamental understanding regarding the manner in which 2D structures are impacted by defects compared to the bulk. Such an approach would therefore serve the scientific community with the materials design tools needed to fabricate the next generation of supercapacitor devices. It must be borne in mind that the way in which carrier transport is enhanced in semiconductors is fundamentally the same, regardless of composition. Defects generate bandgap states that either generate electrons in the conduction band or holes in the valence band. Therefore, we believe that the discussion, based on experimental results, of the magnitude of this effect for 2D and 3D materials will be of utmost benefit to the interested community.

## Review

### ZnO as electrode material for supercapacitors

Zinc oxide (ZnO) is a highly defective semiconductor material, regardless of its synthesis route, that has a large bandgap energy (*E*_g_) at room temperature. However, defect types, their locations, and their concentration depend on two major characteristic properties, namely size and morphology. To detect, identify, and determine the defect structures and their concentrations microscopic characterization techniques need to be used to yield detailed information on the local environment of the lattice atoms and defects.

With the aid of advanced characterization techniques one may get valuable information on site symmetry, atomic bonding, and, in particular, on the bandgap energy of semiconductors. Raman, photoluminescence (PL), UV–vis, and electron paramagnetic resonance (EPR) spectroscopy techniques among the most powerful techniques to extract detailed information on the defect structures of ZnO. Also, electrical impedance spectroscopy (EIS) and cyclic voltammetry (CV) are sensitive to electrical properties such as specific capacitance and impedance, which can be correlated with the existence of the defects. Owing to the extreme sensitivity of EPR spectroscopy (10^11^ spins/g) to paramagnetically active defect centers, one may correlate the information on the local electronic configuration from EPR spectra with Raman and PL spectra. Thus, when such semiconductor materials are used as electrode materials in storage devices, for example, supercapacitors, the device performance can be enhanced substantially. This enhancement can be correlated to materials characteristics, such as nanoscale size effects, doping, the synergy between the defective electrode and the counter carbonaceous electrode, and as well as the existence of defects.

**Figure 1:**
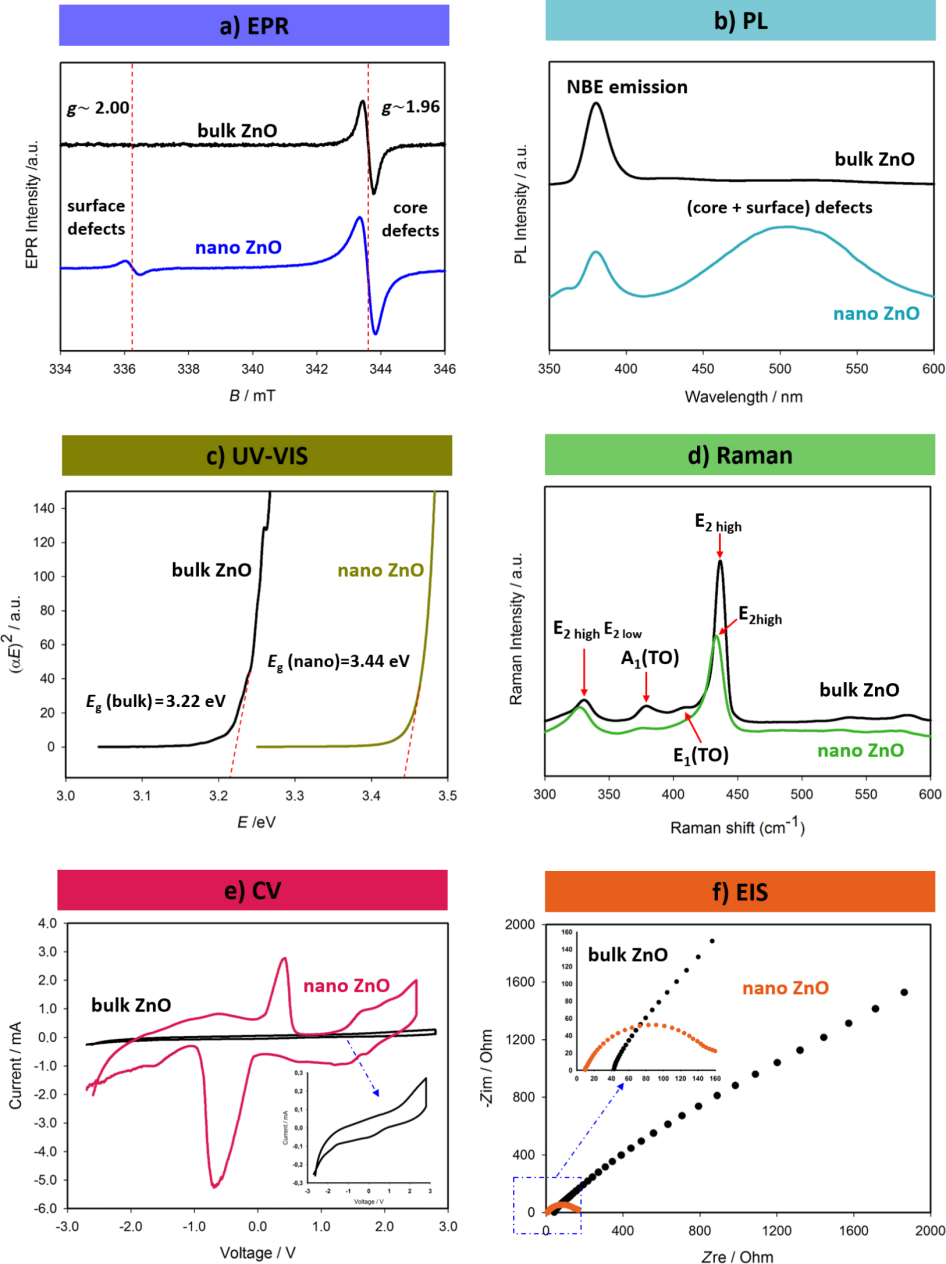
Characteristic results for bulk and nanometer-sized ZnO regarding the analysis of their intrinsic point defects and the performance as electrode in supercapacitor devices. (a) X-band EPR spectra indicating two distinct signals with *g*-factors of 1.96 and 2.00 arising from the core and from surface defects, respectively [1–6]. These are paramagnetic defects, which are mainly singly or doubly ionized oxygen vacancies located either in volume or at the surface of the material. (b) PL spectra showing all possible defect centers, including non-paramagnetic ones. The defects emit visible light yielding a broad emission band [4]. (c) Tauc plot obtained from UV–vis reflectance measurements. Nanometer-sized ZnO shows a significant increase in bandgap due to quantum confinement effects [7–9]. (d) Structural changes of ZnO during the size reduction from bulk to the nanoscale can be tracked via Raman spectroscopy. Nanoscale ZnO exhibits significant blueshift, broadening, and softening of the vibration modes in the fingerprint E2 (high) mode, the second-order Raman E2(high) − E2(low) difference mode, and the transverse optical A1 and E1 modes [3,10]. (e) Intercalation–deintercalation (charge–discharge) behavior measured using CV. The results indicate that at when ZnO has a small crystallite size, the working mechanism of the supercapacitor changes from an electric double-layer capacitor (EDLC) to a Faradaic capacitor yielding higher specific capacitance values. Here, the supercapacitor device consists of four main components: ZnO (electrode 1), graphene foam (electrode 2), glass fiber as separator and, 1 M LiPF_6_ in a 1:1 mixture of ethylene carbonate and diethyl carbonate as electrolyte [7,10–11]. (f) Nyquist plot of a supercapacitor obtained via EIS indicating a decrease in resistance with an increase of surface-defect concentration at the nanoscale [7].

Over the years, we gained a lot of experience in the defect characterization of ZnO both in bulk and at the nanoscale. Figure 1 gives a summary including all the abovementioned characterization techniques for bulk and nanoscale ZnO. A direct comparison of the characteristics of bulk and nanoscale ZnO fits very well to the core–shell model described elsewhere [1–3].

In particular, EPR spectroscopy and PL spectroscopy (Figure 1a,b) are important for the investigation of defects here. Both techniques reveal distinct signals that originate from point defects. PL yields defect-related emission from all possible defects whereas EPR reveals only signals from paramagnetic defects [7]. Therefore, using EPR one obtains signals from the vacancies or interstitials that are ionized and become paramagnetic. In ZnO, singly ionized oxygen vacancies are the major defect centers and give an EPR signal around g ≈ 1.96 [2–5]. Compared to *g*_e_ ≈ 2.0023, this resonance requires a higher magnetic field and, thus, a higher microwave frequency. In other words, higher microwave and Zeeman energy are required for this kind of allowed electronic transition. This is mainly due to the strong spin–orbit coupling. According to the core–shell model, the *g* ≈ 1.96 signal arises from the core, where the electrons are trapped and become bound [3,5]. However, defects at the surface require less energy to become unbound and, thus, yield an EPR signal around *g* ≈ 2.004, which is very close to the value of *g* of free electrons. The surface defects accumulated in the shell of the nanocrystal give a value of *g* similar to that of free electrons due to their delocalization [2–3,5]. This information can only be obtained via EPR spectroscopy. The intensity of the surface-defect signal, in general, increases when the mean crystallite size is reduced because of the changing surface-to-volume ratio.

In contrast, PL spectroscopy is highly sensitive to two kinds of emission. One is due to electron–hole (e–h) recombination, which is related to the bandgap energy [4]. Indeed, this emission has nothing to do with the defects. In semiconductors; one may obtain a hint about the value of *E*_g_. For instance, for bulk ZnO, the emission due to the e–h recombination appears in the UV region around 380 nm, which corresponds to ca. 3.2 eV. Hence, this emission is also called near-band-edge (NBE) emission [4–5]. Intrinsic point defects reveal a broadband PL emission in the visible-light region. Here, the situation is a little complicated due to the inhomogeneous distribution of this visible-light emission. In general, in the case of defective ZnO, the emission has a maximum in the range of green light. But some defect centers have either higher or lower emission wavelengths, in other words, their formation energy might be smaller or higher than that of the ones emitting green light. Therefore, a broad and inhomogeneously distributed PL emission band occurs. This feature becomes more distinct and intense when measuring nanoscale ZnO. It also suppresses the NBE emission [7]. The general approach to identify the defect centers in ZnO via PL, which might also be misleading, is as follows: Of the defect-related PL data in the visible-light range, the broad signal is deconvoluted into several peaks and each maximum of these peaks is assigned to one type of emission. Sometimes the emission energies are matched with defect formation energies calculated using first-principle density functional theory. This approach has many problematic issues. First, of all, synthesis routes play a vital role in the creation of the defect centers. Different routes may create different size distributions. The defects may have different thermodynamics and kinetics. Also, secondary phases may occur, and impurities and morphology may change. All these facts affect the creation of defects. Therefore, the deconvolution of PL spectra cannot yield a unique defect emission model for ZnO. Second, the locations of defect structures are not easily distinguishable using PL spectroscopy. As a result, the combination of PL and EPR is a very powerful combination to get detailed information. Using EPR, one may at least separate the surface and core defects safely.

There are other useful characterization techniques, such as UV–vis spectroscopy, with which one may correlate the size effects and defect concentrations with the change in *E*_g_. This is sometimes called bandgap engineering or tuning of the band gap in semiconductors. However, this approach has major problems. It is semi-empiric and indirect via the Tauc formula [12]. Therefore, it contains highly erroneous data. Here, the approach is to measure the reflectance data and accordingly plot the Tauc formula as a function of the energy. Typical plots of the Tauc formula from UV–vis reflectance spectra are given in Figure 1c. The intersection point at the energy axis gives the value of *E*_g_. However, the determination of the intersection point is not very accurate. According to Figure 1c, defective nanoscale ZnO has a higher value of *E*_g_, which is expected due to the quantum confinement effect [13].

Another crucially important optical characterization technique for the investigation of defects is Raman spectroscopy. The phonon vibration modes are highly sensitive to the existence of point defects, which are reflected in distinct spectral changes in the Raman spectrum. In particular, this method is used for carbon materials to detect the so-called D-band, which belongs to sp^3^-hybridized carbon. For the occurrence of the D-band either C dangling bonds on the surface, C interstitials, or C vacancies need to exist. Raman spectra of metal oxides are more complicated than those of carbon materials because there are many peaks for each mode. The most prominent peak in Raman spectra of ZnO is E_2_(high) for ZnO crystals. Once the size of the ZnO crystallites is below 50 nm, broadening, softening, and a significant shift is observed for this mode, which is indicative of a large amount of defect centers. The existence of defect centers affects the vibration mode and eventually causes a blueshift, as shown in Figure 1d.

Finally, the electrical properties obtained via CV and EIS can also be correlated with the defect structures when the semiconductor metal oxide, here ZnO, is processed chemically or physically as an electrode. This is rather complicated and it is important which method is used to test the electrical properties. The common method is the three-point method in which the material is tested with a counter and a reference electrode in a liquid electrolyte. Here, an electrical device design is not necessarily required and the results are relatively straightforward. In contrast, in the two- or four-point methods, a device, such as a supercapacitor or a battery, is produced and the electrode is tested in interaction with second carbonaceous electrode, electrolyte, and separator. Here, the selection of the materials is crucially important and state-of-the-art materials science and engineering designs determine the energy and power density of the devices. Today, it is an important research goal to find supercapacitor devices with high capacity and high energy density so that in the near future supercapacitors might work together with batteries as an integrated energy storage system. Metal oxides, MXenes, and perovskites are the most promising electrode materials for this end. However, the specific capacitance values of those electrodes are still too low compared to carbonaceous electrodes. Nanoscale dimensions, the increase of the surface-defect concentration, and metal-ion doping may play a vital role in increasing specific capacitance values as well as cycle life and in mitigating degradation problems.

Recently, valuable information has been obtained from the electrical analysis of bulk and nanoscale ZnO as electrode material in supercapacitor devices [7,10–11]. Bulk ZnO exhibits typical electric double-layer capacitor (EDLC) character. Using nanoscale ZnO, however, due to the effect of the surface defects, the device shows pseudocapacitive behavior in which Faradaic interactions are dominating [7]. The Faradaic electrochemical reactions have typical CV plots with additional humps, which can be seen in Figure 1e. These humps, in general, occur due to additional charges, which cause a rise in specific capacitance and energy density and enable the device to act as a battery. Thus one may call such devices “superbat” (battery-like supercapacitors) [11]. To measure the impedance of the electrodes and, hence, the device, an AC voltage is applied and the real and the imaginary part of the impedance are plotted. Such a plot is called Nyquist plot and the first semicircle of the Nyquist plot gives the resistance values. In general, defective and nanoscale ZnO has lower resistance values and the semicircle becomes more prominent, as depicted in Figure 1f. Metal oxide electrodes normally show resistive, rather than conductive, behavior. Therefore, different electrical components contribute to the equivalent circuit of the EIS results. Warburg element, charge transfer resistance (*R*_ct_), and equivalent series resistance (ESR) are some of the elements that contribute to the resistive behavior [11]. At this point, we highly suggest to the reader to see our recent mini-review about the current progress and future trends in materials development for supercapacitors [8].

It is important to point out that in terms of energy-related applications, the use of metal oxides is rather limited. An enhancement of the devices can only be achieved by controlling size, bandgap, doping, defect structures, and morphology. Thus, energy density and specific capacitance of metal oxides almost reached the maximum (theoretical) values. Novel types of materials that are an alternative to metal oxides should be studied intensively. One of the most promising alternatives to metal oxides are two-dimensional (2D) MXenes. Their properties include environmental sustainability, lamellar structure, high transformation efficiency, high surface hydrophilicity, and good electrical conductivity [14]. Therefore, in the following section we discuss the main properties of MXenes as electrode material for their use in supercapacitor devices.

### MXenes as electrode material in supercapacitors

Today, there is a great interest in renewable energy sources, such as solar and wind, for various reasons, such as the depletion of fossil fuels and the smaller environmental impact [15–17]. The need of green and clean energy comes with the price of new energy storage systems where the most prominent challenge lies within the integration of sources [15,18–19]. Indeed, there is a need for quickly responding systems in wind and solar energy facilities [15]. Also, energy storage systems are not only required for renewable energy sources but also for portable or wearable electronics and electric vehicles [16,20–21]. To tackle these challenges supercapacitors are amongst the numerous candidates, due to unique properties such as high power density, fast charge/discharge capability, long cycling stability, as well as environmental sustainability [15–18,21–22]. Unfortunately, supercapacitors are not fully in the market due to manufacturing cost, stability, and lifetime concerns [15].

Electrodes and electrolytes are two key parameters that affect the design of the supercapacitors [15]. Until now, carbon (high surface area), metal oxides (high specific capacitance and low resistance), and conducting polymers are used as electrode materials. The issues of cost, stability, and lifetime are not resolved yet [15]. Graphene seems like one of the possible main electrode materials because of its unique properties, such as ultrathin structure and heterojunction behavior [15].

In the search of other 2D materials, MXenes, which are a novel class of 2D metal carbides, were discovered at Drexel University in 2011 during research in which MAX phases were used as electrode materials in batteries [15,20–21,23]. Gogotsi et al. [24–26], who are the pioneers of MXene materials, have defined the relation to MAX phases in a very clear way: MXenes can be produced by etching the A layer from MAX phases. The suffix “ene” is added to emphasize the similarity to graphene. MAX phases are a large family of hexagonal layered ternary transition-metal carbides, carbonitrides, and nitrides with the formula: M*_n_*_+1_AX*_n_* where M denotes a transition metal (Sc, Ti, V, Cr, Zr, Nb, Mo, Hf, or Ta), A denotes a group-13 or group-14 element (Al, Si, P, S, Ga, Ge, As, Cd, Ln, Sn, Tl, or Pb), and X denotes carbon and/or nitrogen [15,18–19,21,23] with *n* = 1–3. In addition, MAX phases and MXenes are conducting ceramics [15,18,27]. Several etching processes have been developed to synthesize particular MXenes. With the results not meeting with expectations, the scientists understood that MAX phases are layered thick solids and, thus, do not contain spaces for the penetration of Li ions into the electrode [15]. Therefore, to enhance the performance of MXene supercapacitors an increased ion transfer rate is needed [18,23]. MXenes exhibit two major changes due to the removal of the A layer from the parental MAX phases [15]. The reason behind removing the A layer, which also paved the way for the discovery of the MXenes, is that MX bonds are comparably stronger than the MA bonds [15]. MA bonds can be broken more easily than MX bonds. Thus, the A layer can be etched to achieve a highly conductive layer structure. After the etching process, MXenes have a great number of active groups, such as –OH and =O, which results in superior hydrophilicity, chemical reactivity, and large contact area. This implies a potential of MXenes for the use in supercapacitors [16,18–19,22–23,28]. Moreover, MXenes can be used, for example, in water purification, as electrochemical actuators, as transparent conductive electrodes, and as biosensors [21–22,28].

To enhance the performance of MXene supercapacitors, a variety of materials, such as graphene and carbon nanotubes (CNTs), tin(IV) oxide (SnO_2_), and iron(III) oxide (Fe_2_O_3_), may be used to increase the layer spacing, which directly increases the ion transfer rate [16–18]. In other words, the intercalation or modification of MXenes helps to prevent the possibility of stacking, increases ion adsorption sites, and enhances electrochemical properties [19,22]. Therefore, MXene-based composites have a promising future [16–17]. MXenes have been shown to be potential electrode materials both for Li-ion batteries and supercapacitors [20–21]. After the initial discovery, different researchers from all around the world have been combining efforts to synthesize MXenes and used them for different applications. In Table 1, we have summarized some of the works in which MXenes were used as electrode material in high-performance supercapacitor applications.

**Table 1:** Summary of MXenes as electrode materials and their electrochemical performance in supercapacitors.

Electrode material	CE or composite^a^	SC type^b^	Electrolyte	Potential Window (V)	Specific Capacitance	Ref.

Ti_3_C_2_T*_x_*	CNTs	—	MgSO_4_	−0.8 to 0.1 V	350 F·cm^−3^ at 2 mV·s^−1^	[20]
Ti_3_C_2_T*_x_*	[B_12_H_12_]^2−^	pseudo	H_2_SO_4_	−0.2 to 0.35 V	366 F·g^−1^ at 2 mV·s^−1^	[18]
wavy Ti_3_C_2_T*_x_*	R60/CNT/PANI	pseudo	H_2_SO_4_	—	116 F·g^−1^ at 10 mV·s^−1^	[27]
dMo_2_CT*_x_*	—	—	H_2_SO_4_	−0.30 to 0.30 V	196 F·g^−1^ at 2 mV·s^−1^	[28]
Ti_3_C_2_T*_x_*	N-CuMe_2_Pc	EDLC and pseudo	H_2_SO_4_	0 to 1 V	786 F·g^−1^	[16]
Ti_3_C_2_T*_x_*	Ti_3_C_2_T*_x_*@N-C	pseudo	KOH	−1 to 0.4 V	260 F·g^−1^	[21]
Ti_3_C_2_T*_x_*	Co(OH)_2_	pseudo	KOH	−0.1 to 0.5 V	153 F·g^−1^	[17]
Ti_3_C_2_T*_x_*	NiS	pseudo	KOH	−0.2 to 0.8 V	857 F·g^−1^	[19]
Ti_3_C_2_T*_x_*	GO	pseudo	H_2_SO_4_	−0.6 to 0.2 V	329 F·g^−1^ at 5 mV·s^−1^	[23]
Ti_3_C_2_T*_x_*	(Ti_3_C_2_T*_x_*/C)	pseudo	KOH	−1 to 0 V	226 F·g^−1^ at 1 A·g^−1^	[22]
Ta_4_C_3_	plain	EDLC	H_2_SO_4_	−0.1 to 1 V	481 F·g^−1^	[15]
Ti_3_C_2_T*_x_*	NiO	asymmetric	KOH	0 to 0.8 V	80 F·g^−1^	[29]
Ti_3_C	—	pseudo	KOH	−0.9 to −0.4 V	117 F·g^−1^	[30]
Ti_3_C_2_	CNT	pseudo	KOH	0.1 to 0.55 V	393 F·cm^−3^	[31]
Ti_3_C_2_	CNT	pseudo	KOH	−0.4 to −0.2 V	85 F·g^−1^	[32]
Ti_2_CT*_x_*	—	pseudo	KOH	0 to 0.7 V	51 F·g^−1^	[33]
Ti_3_C_2_T*_x_*	GO	pseudo	H_2_SO_4_	−0.7 to 0.3 V	1445 F·cm^−3^	[34]
Ti_3_C_2_T*_x_*	—	pseudo	H_2_SO_4_ and PVA	0 to 0.6 V	23 mF·cm^−2^	[35]
Ti_3_C_2_T*_x_*	—	pseudo	H_2_SO_4_	−0.6 to −0.1 V	380 F·g^−1^	[36]
Ti_3_C_2_T*_x_*	rGO	pseudo	KOH	−1 to −0.3 V	405 F·g^−1^	[37]

^a^CE or composite: Here, CE stands for counter electrode. In supercapacitor research, MXene materials are used either as electrode or as the part of a composite electrode. ^b^SC stands for supercapacitor.

Syamsai et al. focused on tantalum carbide (TaC*_x_*), which has advantageous characteristics, such as biocompatibility, photothermal efficiency, low Seebeck coefficient, as well as good conductivity. They synthesized tantalum carbide MXene sheets from a tantalum aluminum carbide (Ta_4_AlC_3_) MAX phase through etching the intermediate aluminium with the aid of hydrofluoric acid (HF). Analysis of the synthesized tantalum carbide MXene sheets was carried out using X-ray diffraction measurements (XRD), field-emission scanning electron microscopy (FE-SEM), transmission electron microscopy (TEM), and Raman spectroscopy. The results of XRD, FE-SEM, and Raman showed that tantalum carbide MXenes are layered solid structures with a hexagonal crystal lattice. Because the aluminum was removed, there is no visible Raman vibration. Moreover, CV, chronopotentiometry (CP), and EIS were used to test the electrochemical behavior of the synthesized tantalum carbide MXenes. In 0.1 M sulfuric acid (H_2_SO_4_) as electrolyte, a maximum specific capacitance of 481 F·g^−1^ was achieved at 5 mV·s^−1^. The results indicate that tantalum carbide MXenes are appropriate for the use in supercapacitor applications [15]. Zhao et al. used an alternative fabrication technique and developed highly flexible sandwich-like MXene/CNT papers. The MXene/CNT papers yielded a volumetric capacitance of 350 F·cm^−3^, which is higher than that of pure MXene, randomly mixed MXene/CNT papers, carbon-based electrodes (60–180 F·cm^−3^), or a graphene-based electrode (260 F·cm^−3^). In this work, they proved that by composing sandwich-like architectures of one-dimensional (1D) and 2D nanomaterials, a good electrochemical performance can be obtained [20]. Li et al. developed dodecaborate/MXene composites. Borate has strong B–B bonds and is an important photoelectric material. Due to its high optical and electrochemical activity, borate is preferred in energy storage applications that offer high power density, stability, and safety. A specific capacitance of 366 F·g^−1^ was achieved at 2 mVs^-1^ [18]. In addition, Li et al. designed an asymmetric pseudosupercapacitor of wavy-Ti_3_C_2_T*_x_*/reduced graphene oxide (rGO)/CNT/polyaniline(PANI), in which the Ti_3_C_2_T*_x_* MXene is used as positive and rGO/CNT/PANI as negative electrode. In this design, both electrodes are pseudocapacitive and compact. Thus, high volumetric capacitances are achievable. The designed wavy-Ti_3_C_2_T*_x_*/rGO/CNT/PANI asymmetric pseudosupercapacitor yielded 116 F·g^−1^ at a scan rate of 10 mV·s^−1^ [27]. Halim et al. synthesized large-scale 2D Mo_2_CT*_x_* from Mo_2_Ga_2_C powder by etching gallium (Ga) selectively with the aid of two etchants, hydrogen fluoride (HF) and lithium fluoride (LiF)/HCl, with subsequent delamination. The morphology of the developed flakes differed with the used etchant. After using LiF/HCl as etchant less defective flakes were obtained. The volumetric capacitance of the delaminated Mo_2_CT*_x_* “paper” was measured as 700 F·cm^−3^ at 2 mV·s^−1^ [28]. Ramachandran et al. developed a MXene composite with non-peripheral octamethyl-substituted copper(II) phthalocyanine, MXene/N-CuMe_2_Pc, with different fractions of N-CuMe_2_Pc nanorods. The sample M10P2, in which 10 mg of MXene and 2 mg N-CuMe_2_Pc were used, exhibited high ionic conductivity and the measured value of the specific capacitance was 786 F·g^−1^ [16]. Li et al. synthesized monolayer wrinkled Ti_3_C_2_T*_x_* grafted with HF and decorated with N-doped carbon by multiple chemical processes. Because the number of active sites is increased, a faster ionic transport is observed. Also, a specific capacitance of 260 F·g^−1^ was measured [21]. Jiang et al. developed a layered Co(OH)_2_/Ti_3_C_2_T*_x_* composite material via growing Co(OH)_2_ nanosheets onto Ti_3_C_2_T*_x_* MXene. The total specific capacitance of the resultant composites was twice as high as that of Co(OH)_2_ nanosheets [17]. Liu et al. synthesized a MXene/nickel sulfide (NiS) nanocomposite using a basic hydrothermal technique and measured a specific capacitance of 857.8 F·g^−1^. NiS has advantages such as good conductivity and abundant raw materials. Also, it is less harmful to the environment [19]. Miao et al. fabricated 3D porous MXene-rGO films by using a self-propagating approach, which involves great enthalpy change, chain reactions, and drastically propagates onto the entire film in seconds. As a result, a specific capacitance of 392.2 F·g^−1^ was measured at 5 mV·s^−1^ from 3D MXene-rGO with 20% graphene [23]. Zhu et al. proposed a way to intercalate organic cations into T_3_C_2_T*_x_* to acquire carbon–MXene heterostructures. Due to intercalation and calcination processes, the interlayer space is increased, which leads to and increased capacitance of 226 F·g^−1^ at 1 A·g^-1^ [22]. Xia et al. used a Ti_3_C_2_T*_x_* MXene electrode decorated with nickel oxide (NiO) nanosheets. The electrode was synthesized via a hydrothermal method. The electrode used in a supercapacitor showed an excellent specific capacitance of 80 F·g^−1^ and an energy density of 1.04 × 10^−2^ Wh·cm^−3^ [29]. Lin et al. synthesized titanium carbide Ti_3_C_2_ through exfoliation of the ternary carbide Ti_3_AlC_2_ and used it as electrode material in a supercapacitor. A maximum capacitance of 117 F·g^−1^ was reported in this research with high cycle stability and good capacitance retention [30]. Yan et al. used a composite material comprising MXene family Ti_3_C and CNTs as an electrode material to enhance the performance of a supercapacitor. They reported a high volumetric capacitance of 393 F·cm^−3^ and increased rate capability, as well as excellent cycling stability [31]. Dall’Agnese et al. showed how the electrochemical performance of a supercapacitor was affected by the architecture and composition of the electrode. The electrode material was made up of a composite of Ti_3_C_2_ along with CNTs. A maximum capacitance value of 85 F·g^−1^ was reported with high rate capability and good cyclability [32]. Rakhi et al. reported that the performance of Ti_2_CT*_x_* MXene electrodes in a supercapacitor device can be enhanced performance by heat treatment of the MXene electrodes. The highest specific capacitance value reported here was 51 F·g^−1^. The sample was annealed in N_2_/H_2_ atmosphere [33]. Fan et al. synthesized MXene/holey graphene (HGO) films, to be used as electrode material in supercapacitors, through the filtration of alkalized MXene and HGO dispersions. The prepared material, when used as a composite film electrode for supercapacitors, showed a high volumetric capacitance of 1445 F·cm^−3^ with enhanced rate capability and high mass loading [34]. Huang et al. reported a comparatively simple spray coating approach to manufacture paper-based solid-state flexible micro-supercapacitors (FMSCs) with a sprayable MXenes as conductive ink. FMSCs are innovative candidates for portable and on-chip energy storage. In this work, using Ti_3_C_2_T*_x_* MXene electrodes further enhanced the performance of the supercapacitor and a high areal capacitance of 23.4 mF·cm^−2^ was reported [35]. Bayram et al. developed an MXene electrode for supercapacitors with tunable architectures. The processing conditions of the MXene aerogel architectures affect the capacitance of the supercapacitor. MXene aerogels with lamellar structures were fabricated through the unidirectional freeze casting of 2D Ti_3_C2T*_x_* aqueous colloidal suspensions. A maximum capacitance of 380 F·g^−1^ was measured with excellent cyclic stability [36]. Xu et al. developed an rGO/Ti_3_C_2_T*_x_* MXene-based supercapacitor. The rGO/Ti_3_C_2_T*_x_* films were synthesized using vacuum-assisted filtration of rGO/Ti_3_C_2_T*_x_*. The supercapacitor showed specific capacitance values up to 405 F·g^−1^. The approach used here eliminated the requirement of a delamination of MXenes and provide a means to develop thick electrodes with good electrolyte accessibility [37].

MXene materials have been successfully shown to possess tremendous potential to be used as electrode material in energy storage devices. The energy storage of MXene material is based primarily on the accommodation of cations between the 2D MXene layers [38]. MXenes with a 2D lamellar structure also have good electric conductivity, hydrophilic surface properties, and they can intercalate different cations between their layers. There is still great room for further research on MXene-related energy storage devices. Currently, different researchers are working to further enhance the performance of such devices [12,39].

## Conclusion

In this short perspective article, two of the electrode materials with the highest potential for supercapacitor applications were discussed, namely ZnO and MXenes. Supercapacitors are gaining high research interest due to the high power density they offer in energy applications. Efforts to increase their energy density are continuously being made so they can substitute batteries for good. Metal oxides are used as electrode materials for quite some time, and ZnO is gradually gaining more popularity. ZnO is an inherently highly defective material with a large bandgap energy at room temperature. It is one the most extensively studied prototype semiconductors regarding defect structures. The intrinsic point defects play a crucial role in device performance regarding sensors, photovoltaics, and energy storage. In this context, supercapacitors are a major application of ZnO as electrode material. This contribution summarizes the results using all possible microscopic characterization techniques to detect ZnO defect structures, their role, and the effect of their concentration. The results given in Figure 1 show that the intrinsic point defects play a crucial role in both bulk and nanoscale ZnO regarding the performance as electrode material in supercapacitor devices. Compared to ZnO, MXenes are quite recently developed materials. However, they are already showing tremendous potential to be used as an electrode material in supercapacitors. The techniques of structural defect characterization in metal oxides can be applied to 2D materials such as MXenes. We are indeed aiming to transfer the knowledge about semiconductors to 2D materials using the two prototype materials ZnO and MXene. Starting investigations of these two prototype materials will ease future developments of more complicated crystal structures or composites.

Finally, we collected numerous outstanding published works of different researchers in which MXenes were used as electrode materials in supercapacitors, whether as pure materials or with other materials in composite form. We presented the maximum specific capacitance values achieved in all those published works, together with the type of supercapacitor used. This can be of great help for people who want to work on MXene for supercapacitor applications. So, as efforts towards new and improved storage devices are continuously made, research on electrode materials for these applications will always remain open for more innovative ideas and techniques.
